# Specific inhibition of ICAM-1 effectively reduces bladder inflammation in a rat model of severe non-bacterial cystitis

**DOI:** 10.1038/srep35672

**Published:** 2016-10-26

**Authors:** Xiang Zhang, Hongchao He, Guoliang Lu, Tianyuan Xu, Liang Qin, Xianjin Wang, Xingwei Jin, Boke Liu, Zhonghua Zhao, Zhoujun Shen, Yuan Shao

**Affiliations:** 1 Department of Urology, Ruijin Hospital, Shanghai Jiaotong University School of Medicine, Shanghai, China; 2 Department of Urology, Ruijin Hospital North, Shanghai Jiaotong University School of Medicine, Shanghai, China; 3 Department of Pathology, School of Basic Medical Sciences, Fudan University, Shanghai, China; 4 Department of Urology, Huashan Hospital, Fudan University, Shanghai, China

## Abstract

The development and progression of bladder pain syndrome/interstitial cystitis (BPS/IC) is closely related to bladder inflammation. Intercellular adhesion molecule 1 (ICAM-1) is associated with bladder inflammation in BPS/IC. We investigated the effect of specific inhibition of ICAM-1 using an anti-ICAM-1 antibody (AIA) on bladder inflammation in a rat model of severe non-bacterial cystitis (NBC) resembling BPS/IC by evaluating the bladder inflammation grade, mast cell infiltration and related cytokines and receptors. We also compared the effects of AIA with the COX-2 inhibitor celecoxib and the neurokinin-1 receptor (NK1R) inhibitor aprepitant. Our NBC model was established by intraperitoneal injection of cyclophosphamide combined with intravesical protamine/lipopolysaccharide, which resulted in severe bladder inflammation and increased mast cell infiltration, similar to the pathological changes of BPS/IC. Inhibition of ICAM-1 by AIA significantly decreased the bladder inflammation grade and mast cell counts, which was accompanied by a reduction of purinergic receptors (P2X2/P2X3), prostaglandin E2, EP1/EP2 receptors, TNF-α, NK1R, and ICAM-1. Moreover, AIA showed superior effects to those of celecoxib and aprepitant treatment in improving the bladder inflammatory response. Our results suggest that ICAM-1 may play a critical role in bladder inflammation in severe NBC and may be used as a novel therapeutic target in non-bacterial bladder inflammation such as BPS/IC.

Bladder pain syndrome/interstitial cystitis (BPS/IC) is characterized by the key symptoms of pelvic and bladder pain (associated with bladder filling and relieved by voiding) accompanied by voiding dysfunction such as urgency, urinary frequency, and nocturia^1^. Histological changes in the bladder tissue of BPS/IC patients include edema and hemorrhage in the submucosa, mast cell invasion, and over-sensitivity of the neural nerve endings^2^. The persistent existence of non-bacterial inflammatory changes in the bladder tissue is thought to be the main reason for the untreated symptoms of clinical BPS/IC patients^3^. Various publications have identified that many inflammatory factors are involved in the progression of BPS/IC, especially in mediating the development of inflammation in the bladder tissue, including purinergic receptors (P2X3 and P2Y2), prostaglandin E2 (PGE2), E-series prostaglandin (EP) receptor subtypes (EP1 and EP2), tumor necrosis factor alpha (TNF-α), intercellular adhesion molecule-1 (ICAM-1), and neurokinin-1 receptor (NK1R)^4^,^5^,^6^,^7^; however, the potential key players in non-bacterial cystitis such as BPS/IC still remain unidentified.

ICAM-1 is a proinflammatory factor that can lead to the release of inflammatory mediators by activating mast cells and leukocyte adhesion to the inflammatory area^8^. Increased ICAM-1 secretion can mediate endothelial cell changes and vascular leakiness, which result in the edema^9^. Enhanced ICAM-1 intensity has been observed in patients with BPS/IC and is associated with the degree of bladder inflammation^7^,^10^. Principal component analysis has identified ICAM-1 as one of three main characteristics that discriminate tissues of IC patients from controls^11^. Furthermore, Leppilahti *et al*. showed that blocking the ICAM-1 receptor might be the pharmacological mechanism by which hyaluronic acid can relieve the symptoms of BPS/IC^12^. All these publications strongly suggest that ICAM-1 might play a vital role in bladder inflammation of BPS/IC.

Thus, we hypothesized that ICAM-1 may act as a key cytokine that regulates the development of BPS/IC. A severe non-bacterial cystitis (NBC) rat model, which was established by intraperitoneal cyclophosphamide (CYP) injection combined with intravesical administration of protamine/lipopolysaccharide (PS/LPS), was used in this study. In our previous report, we demonstrated that this NBC rat model was more suitable than other models that use intraperitoneal CYP or intravesical PS/LPS alone to mimic bladder lesions of BPS/IC patients^10^. Using this NBC model, we investigated the effect of blocking ICAM-1 with a specific anti-ICAM-1 antibody on bladder inflammation and compared its efficacy with celecoxib and aprepitant.

## Results

### The rat model induced by CYP and PS/LPS

**Figure 1:**
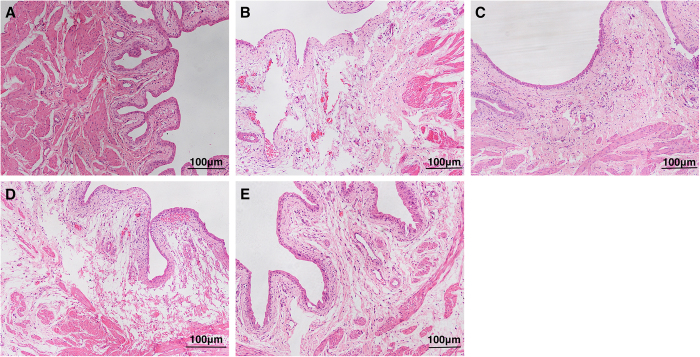
Bladder inflammatory changes in rats. HE stain, x100 magnification. (**A**) Normal control rats (group 1) had no edema or inflammation. (**B**) Cystitis model rats (group 2) had obvious inflammation, including extensive submucosa edema and marked microhemorrhage accompanied by a significantly thinner urothelium. (**C**) Model + celecoxib rats (group 3) had severe vascular proliferation, microhemorrhage, and errhysis with edema of the submucosa. (**D**) Model + aprepitant rats (group 4) showed slight congestion of the microangium and severe congestion of the submucosa. (**E**) Model + AIA rats (group 5) had no obvious changes except for slight edema.

**Figure 2:**
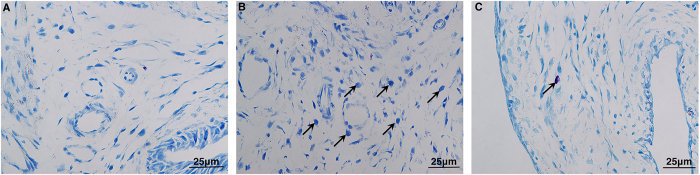
Distribution of mast cells (arrows). Toluidine blue stain, x400 magnification. (**A**) Control group. No obvious mast cells were observed. (**B**) Cystitis model group. Several mast cells were observed in the mucosa of the bladder tissue. (**C**) Model + AIA group. Only one mast cell was detected. AIA: anti-ICAM-1 antibody.

The presence of bladder inflammation and mast cell counts were assessed by visual inspection of bladder morphology in HE-stained and toluidine blue-stained samples, respectively. Consistent with our previous study, the NBC model induced by intraperitoneal CYP combined with intravesical PS/LPS exhibited profound inflammation, including vascular congestion, microhemorrhage, extensive submucosa edema, and mast cell infiltration (Figs 1B and 2B and Table 1). Moreover, the expression levels of P2X3 and P2Y2 receptors, PGE2, EP1/EP2 receptor, TNF-α, NK1R, and ICAM-1 were significantly increased in the NBC model group (Table 2).

### Effect of different interventions on bladder inflammation and mast cell infiltration

When the NBC rats in group 3 were treated with celecoxib, we observed only one or two cell layers of mucosa, and complete denudation of the urothelium was not noted. We also observed obvious edema in the submucosa area and rich capillaries accompanied by congestion and diapedesis of erythrocytes (Fig. 1C). Compared to the model group or group 3, the severity of hemorrhage and congestion in group 4 (administered aprepitant) were clearly decreased (Fig. 1D). In group 5 (NBC rats administered AIA), the bladder tissue exhibited less obvious morphological changes than groups 2, 3, and 4, except for slight edema and a thicker epithelium (Fig. 1E).

Our NBC model induced by intraperitoneal CYP with intravesical injection of PS/LPS exhibited a higher grade of bladder inflammation and greater mast cell counts than normal rats (Table 1). These effects were remarkably inhibited by aprepitant and AIA (Table 1). Moreover, AIA showed better inhibitory efficacy on bladder inflammation and mast cell infiltration than celecoxib treatment and reduced mast cell counts more than aprepitant treatment (Table 1).

### Effect of different interventions on cytokines and receptors

Table 2 shows the differences of each receptor and cytokine in the various groups.

The NBC model had significantly higher levels of measured cytokines and receptors (P2X3 and P2Y2 receptors, PGE2, EP1/EP2 receptor, TNF-α, NK1R, and ICAM-1) than the normal control group. After celecoxib treatment, the levels of P2X3/P2Y2 receptors, PGE2, EP1/EP2 receptors, NK1R, and TNF-α were significantly decreased, but similar results were not observed after ICAM-1 treatment (Table 2). In group 4, aprepitant treatment decreased the expression of all cytokines and receptors except for P2X3.

In group 5, AIA treatment resulted in a remarkable reduction of the expression of P2X3/P2Y2 receptors, PGE2, EP1/EP2 receptor, NK1R, TNF-α, and ICAM-1 in the NBC model rats. AIA treatment resulted in a greater decrease in the expression of PGE2, EP1/EP2 receptor, TNF-α, and NK1R than celecoxib treatment. The inhibitory effect of AIA on P2X3, P2Y2, and EP1/EP2 receptors and NK1R was remarkably stronger than that of aprepitant (Table 2). Furthermore, strong positive correlations were observed between bladder inflammation grade and mast cell counts with the ICAM-1 level (Spearman correlation coefficients, 0.767 and 0.789, respectively, P < 0.001) among all 5 groups.

## Discussion

In the present study, we investigated the involvement of ICAM-1 in bladder inflammation in an NBC rat model, which shows similar inflammatory characteristics to IC. We found that the specific inhibition of ICAM-1 significantly attenuated bladder inflammation and mast cell infiltration, accompanied by a reduction of related inflammatory cytokines and receptors (P2X3 and P2Y2 receptors, PGE2, EP1/EP2 receptor, NK1R, TNF-α, and ICAM-1). In addition, our results showed that the ICAM-1 level was positively correlated with the bladder inflammation grade and mast cell counts of the bladder tissue. These results suggest that increased ICAM-1 expression may be associated with bladder inflammation and the development of NBC.

Several studies have reported that the main pathological changes in the bladder tissue of BPS/IC patients include denudation of the urothelium, mast cell infiltration, and edema in the lamina propria with stromal hemorrhage and congested venules^13^,^14^. Moreover, increased expression levels of P2X3 and P2Y2 receptors, PGE2, EP1/EP2 receptor, TNF-α, NK1R, and ICAM-1 in bladder tissue were found in BPS/IC patients^4^,^5^,^6^,^7^. Based on immunohistochemical observations, our NBC model showed more severe edema, hemorrhage, vasodilation, and mast cell infiltration in the bladder tissue than the control group. Furthermore, the expression levels of P2X3 and P2Y2 receptors, PGE2, EP1/EP2 receptors, TNF-α, NK1R, and ICAM-1 in our model group were significantly higher than those in normal controls. Therefore, the NBC model created in our study had similar pathological characteristics to the bladder tissue of BPS/IC patients.

PGE2 is synthesized from arachidonic acid through the cyclooxygenase-2 (COX-2) pathway and has been demonstrated to participate in the development of inflammation via the EP receptor subtypes EP1 and EP2^15^. Wada N *et al*. have shown that the expression levels of PGE2 and EP1/2 mRNA are significantly higher in BPS/IC patients^4^. Several studies have also suggested that the PGE2/EP1/EP2 pathway is involved in the development of BPS/IC^4^,^16^. Importantly, the results of Takahashi’s study showed that PGE2 can regulate the expression of ICAM-1 via the EP2 receptor^17^. In this study, oral administration of celecoxib, which can inhibit the transformation of arachidonic acid to PGE2, significantly decreased the expression of PGE2, EP1/EP2 and P2X3/P2Y2 receptors, NK1R, and TNF-α; however, celecoxib had no effect on inflammation grade. AIA treatment showed a greater inhibitory effect on the severity of bladder inflammation and mast cell counts than celecoxib. The expression levels of PGE2, EP1/EP2 receptors, NK1R, and TNF-α in AIA-treated NBC rats were also lower than those of celecoxib-treated rats. These results indicate that ICAM-1 inhibition provides greater therapeutic effects on bladder inflammation than celecoxib.

We also found increased expression of NK1R in the bladder of NBC rats. Sanchez *et al*. reported that NK1R, located in both the urothelium and submucosal layer, plays an important role in the neuroinflammatory response in the bladder^18^. Several studies have shown that the function of NK1R in the urothelium and blood vessels is closely associated with the inflammatory response, and NK1R antagonists inhibit the reduction of urothelial capacitance and inflammatory plasma leakage in several animal models^19^,^20^. Mast cells mediate bladder inflammation through NK1R^21^, and the activation of NK1R increases the expression of ICAM-1 and induces leukocyte infiltration, further supporting the critical role of NK1R in inflammation^22^. Aprepitant mediates its effect by blocking NK1R and has been used to control female overactive bladder^23^. We observed that the inflammation grade and mast cell counts in NBC rats treated with aprepitant were significantly decreased compared to the pure NBC model. Aprepitant treatment exhibited an inhibitory effect on the expression of related cytokines and receptors, except for P2X3; however, the inhibitory effects of AIA on mast cell counts and the expression of P2X3, P2Y2, PGE2, EP1/EP2, and NK1R in the NBC model were all significantly greater than those in aprepitant-treated animals.

P2X3 and P2Y2 receptors and TNF-α are involved in the development of PBS/IC^5^,^6^,^10^. The P2X3 receptor is expressed on endothelial cells and plays an important role in regulating the expression of ICAM-1 and physiological inflammation^24^. TNF-α has been found at a high levels in BPS/IC patient biopsies and can up-regulate the expression of ICAM-1 in urothelial cells^25^. In our study, the direct inhibition of ICAM-1 significantly decreased the expression of P2X3, P2Y2, and TNF-α. Therefore, we hypothesize that specific ICAM-1 inhibition might provide better therapeutic efficacy for bladder inflammation than a COX-2 inhibitor or NK1R antagonist.

The limitations of our research include the small number of animals and measurements of limited inflammatory factors, which may not include all of the signaling pathways of ICAM-1. Future studies should focus on the long-term efficacy of the inhibition of ICAM-1 on the pathophysiological changes of bladder inflammation with respect to all involved signaling pathways in NBC rats. Overall, our study strongly suggests that the specific inhibition of ICAM-1 controls the development of bladder inflammation in NBC rats and provides greater inhibitory effects than celecoxib and aprepitant treatment. These results suggest a vital role of ICAM-1 in the pathophysiological development of bladder inflammation in NBC. ICAM-1 may be further investigated as a novel therapeutic target for the treatment of non-bacterial cystitis, such as BPS/IC.

## Methods

### NBC rat model and interventions

Female Sprague–Dawley rats (250–300 g) were housed in a temperature- and light-controlled room with a 12-hour light–dark cycle. Standard tap water and mouse chow were available for all animals ad libitum. The rats were anesthetized with sodium pentobarbitone (3%, 1.5 ml/kg, intraperitoneal injection) for subsequent production of the NBC model resembling BPS/IC. All experimental procedures were approved by the Institutional Animal Care and Use Committee of Shanghai Jiaotong University School of Medicine and were performed in strict accordance with the National Institutes of Health Guide for the Care and Use of Laboratory Animals (NIH Publication No. 85–23, revised 2011).

The rats subjected to intraperitoneal injection of CYP combined with intravesical PS/LPS were used as the NBC model to represent BPS/IC. During the first day, CYP was administered at 150 mg/kg intraperitoneally. Protamine (0.5 ml, 30 mg/ml, Sigma, St. Louis, MO) was instilled intravesically via a 4 Fr epidural catheter at a slow rate. After 30 min, the bladder was drained and flushed with 0.5 ml of 9% saline. Subsequently, 0.5 ml of LPS (Sigma, St. Louis, MO, 2 mg/ml) was instilled intravesically and maintained for 45 min. All the above procedures were repeated on the third day, as described in our previous study^10^.

The experimental rats were divided into 5 groups. Group 1 served as the normal control; 7 rats received only intravesical instillation of 0.5 ml of saline on the first and third day. Group 2 consisted of 9 NBC model rats. In group 3 and 4, 10 NBC rats were treated with celecoxib (10 mg/kg, Pfizer Co., Ltd) or aprepitant (10 mg/kg, Merck Co., Ltd) through gavage once daily; In group 5, 10 NBC rats were intravesically perfused with anti-ICAM-1 antibody (AIA, 0.1 ml, 0.5 mg/ml, Abcam, ab25375) one hour after PS/LPS instillation on the first and third day. All rats were sacrificed, and the bladders were removed 4 days after the initial manipulations.

### Histological analyses

Each bladder was divided into two equivalent portions. One half was fixed for 72 hours in buffered formaldehyde, embedded in paraffin, and sectioned at 5 μm. Hematoxylin and eosin (HE) stain was used for morphological assessment of the bladder urothelial layer, including the destruction of the urothelium, vessels of the bladder urothelium, leukocyte infiltration, and edema. Toluidine blue staining was used for mast cell counting. The other half of the bladder was stored at −80 °C for measurement of purinergic receptors (P2X3 and P2Y2), EP1/EP2 receptors, NK1R, PGE2, TNF-α, and ICAM-1.

According to a histological examination, the degree of bladder inflammation was classified using a 4-grade scale based on chronic edema, epithelial thinning, petechial hemorrhage, cell infiltration, and desquamation of the uroepithelium. Grade 1 corresponded to the normal control. Grade 2 consisted of simple edema. Grade 3 consisted of edema combined with epithelial layer cleavage and thinning, resulting in mucosal abrasion and the beginning of polynuclear leucocyte infiltration representing mild cystitis. Grade 4 consisted of increased severity and spread of all the above signs plus petechial hemorrhage (complete cystitis)^6^. Mast cells were counted in 10 cross-sections at 400x magnification in the most infiltrated area. All histological evaluations were conducted by two separate pathologists.

### Cytokine and receptor analysis by ELISA

After the tissue was dissolved, the bladder was ground and distributed into two parts. One part was washed with PBS (pH = 7.4), mixed well in a homogenizer and centrifuged at 2500 rpm for 20 min. Then, the supernatant was carefully collected for the analysis of all cytokines and receptors except for ICAM-1 and PGE2. Fifty microliters from each sample was used for duplicate analysis of cytokines and receptors using the rat P2X2, P2X3, EP1, EP2, TNF-α, and NK1R ELISA kits (Biosource International, Inc., CA, USA). The other aliquot was cultured for 12 hours to detect ICAM-1 and PGE2 expression on the cell surface via the solute in the medium. This medium was processed in duplicate using the rat PGE2 (Cayman, Chemical) and ICAM-1 ELISA kits (Biosource International, Inc., CA, USA)^7^ following the manufacturer’s instructions.

### Statistical analysis

The statistical analysis was performed using SPSS 20. A Mann-Whitney U test was used to analyze non-parametric data, such as inflammation grade and mast cell counts. Two-way ANOVA followed by Dunnett’s T multiple comparison test were performed to analyze parametric data. A Spearman test was conducted to analyze the correlation of inflammation grade and mast cell infiltration with the ICAM-1 level. The data are expressed as the means ± standard error (M ± SE), and a value of P < 0.05 was considered statistically significant.

## Additional Information

**How to cite this article**: Zhang, X. *et al*. Specific inhibition of ICAM-1 effectively reduces bladder inflammation in a rat model of severe non-bacterial cystitis. *Sci. Rep.* **6**, 35672; doi: 10.1038/srep35672 (2016).

**Publisher’s note:** Springer Nature remains neutral with regard to jurisdictional claims in published maps and institutional affiliations.

## Figures and Tables

**Table 1:** Bladder Inflammation Grade and Mast Cell Counts.

Bladder Inflammation Grade	Control (7)	Model (9)		Model + Clb (10)			Model + Apt (10)			Model + AIA (10)				
		Grade	P vs Control	Grade	P vs Control	P vs Model	Grade	P vs Control	P vs Model	Grade	P vs Control	P vs Model	P vs Clb	P vs Apt
1	7	0	< 0.001	0	<0.001	0.079	0	<0.001	0.002	2	0.005	<0.001	<0.001	0.123
2	0	0		0			5			6				
3	0	2		7			4			2				
4	0	7		3			1			0				
Mast cell	1 (0–1)	5 (3–7)	<0.001	4 (3–5)	<0.001	0.028	3 (2–4)	<0.001	0.001	2 (1–3)	0.007	<0.001	<0.001	0.019

The data are presented as the median with ranges in parentheses. The Mann-Whitney U test was used to compare nonparametric variables and pairwise differences. A value of P < 0.05 was considered a significant difference. The three intervention groups (Celecoxib, Aprepitant, and AIA) represent NBC rats that received each drug. Clb: Celecoxib; Apt: Aprepitant; AIA: Anti-ICAM-1 antibody Table 2. Cytokines and Receptors.

**Table 2:** Cytokines and Receptors.

Cytokine or Receptor	Control (7)	Model (9)		Model + Clb (10)			Model + Apt (10)			Model + AIA (10)				
	M ± SE	M ± SE	P vs Control	M ± SE	P vs Control	P vs Model	M ± SE	P vs Control	P vs Model	M ± SE	P vs Control	P vs Model	P vs Clb	P vs Apt
P2X3 (ng/ml)	42.11 ± 7.21	280.87 ± 0.17	<0.001	182.74 ± 21.32	0.001	0.011	238.57 ± 21.27	<0.001	0.459	140.46 ± 18.09	0.003	<0.001	0.745	0.024
P2Y2 (ng/ml)	93.47 ± 0.14	354.70 ± 20.75	<0.001	169.40 ± 12.45	0.002	<0.001	188.12 ± 0.15	<0.001	<0.001	122.33 ± 14.12	0.429	<0.001	0.183	0.010
PGE2 (ng/ml)	15.06 ± 0.02	160.35 ± 19.78	<0.001	51.29 ± 4.61	<0.001	0.004	30.19 ± 0.01	<0.001	0.001	18.80 ± 0.79	0.009	0.001	<0.001	<0.001
EP1R (ng/ml)	7.30 ± 0.17	79.75 ± 9.44	<0.001	26.17 ± 2.66	0.001	0.003	16.28 ± 0.01	<0.001	0.003	11.59 ± 0.65	0.001	0.001	0.003	<0.001
EP2R (ng/ml)	5.04 ± 0.01	80.21 ± 9.99	<0.001	30.58 ± 4.87	0.005	0.007	20.04 ± 0.01	<0.001	0.006	8.54 ± 1.50	0.296	0.001	0.011	<0.001
TNF-α (ng/ml)	10.83 ± 0.16	123.14 ± 15.99	<0.001	51.30 ± 14.82	<0.001	0.040	28.53 ± 2.78	<0.001	0.003	21.82 ± 0.84	<0.001	0.002	0.046	0.292
NK1R (ng/ml)	15.42 ± 0.10	77.36 ± 7.97	<0.001	32.30 ± 1.85	0.001	0.003	32.97 ± 0.19	<0.001	0.004	21.99 ± 1.43	0.011	0.001	0.004	<0.001
ICAM-1 (pg/ml)	40.28 ± 5.76	334.18 ± 41.78	<0.001	201.37 ± 20.16	<0.001	0.118	150.38 ± 16.30	<0.001	0.017	125.06 ± 15.98	0.004	0.007	0.076	0.942

The data are presented as the means ± standard error (M ± SE). Two-way ANOVA, followed by Dunnett’s T multiple comparison test, was conducted to analyze parametric data. A value of P < 0.05 was considered a significant difference. The three intervention groups (Celecoxib, Aprepitant and AIA) represent NBC rats that received each drug. Clb: Celecoxib; Apt: Aprepitant; AIA: Anti-ICAM-1 antibody.
